# EXTRACOLONIC FINDINGS—IDENTIFICATION AT LOW-DOSE CTC

**DOI:** 10.1093/rpd/ncab054

**Published:** 2021-04-14

**Authors:** Fredrik Thorén, Åse A Johnsson, Mikael Hellström, Magnus Båth

**Affiliations:** Department of Radiology, Institute of Clinical Sciences, Sahlgrenska Academy, University of Gothenburg, SE-413 45 Gothenburg, Sweden; Department of Radiology, Sahlgrenska University Hospital, SE-413 45 Gothenburg, Sweden; Department of Radiology, Institute of Clinical Sciences, Sahlgrenska Academy, University of Gothenburg, SE-413 45 Gothenburg, Sweden; Department of Radiology, Sahlgrenska University Hospital, SE-413 45 Gothenburg, Sweden; Department of Radiology, Institute of Clinical Sciences, Sahlgrenska Academy, University of Gothenburg, SE-413 45 Gothenburg, Sweden; Department of Radiology, Sahlgrenska University Hospital, SE-413 45 Gothenburg, Sweden; Department of Radiation Physics, Institute of Clinical Sciences, Sahlgrenska Academy, University of Gothenburg, SE-413 45 Gothenburg, Sweden; Department of Medical Physics and Biomedical Engineering, Sahlgrenska University Hospital, SE-413 45 Gothenburg, Sweden

## Abstract

In contrast to optical colonoscopy, computed tomography colonography (CTC) has the ability to reveal pathology outside of the colon. While identification of colorectal lesions at CTC requires only limited radiation dose, the detection of abnormalities in extracolonic soft tissue requires more radiation. The purpose of this study was to investigate the influence of ultra-low-dose (ULD) CTC on the detection and characterisation of extracolonic findings. In a prospective study 49 patients with colorectal symptoms were examined with CTC adding a ULD series (mean effective dose 0.9 ± 0.4 mSv) to the normal unenhanced standard dose (SD) series (mean effective dose 3.6 ± 1.2 mSv). Five radiologists individually and blindly evaluated the ULD, followed by evaluation of the SD after ≥9 weeks (median 35 weeks). A ViewDEX-based examination protocol was used, including a confidence scale and a graded assessment of need for follow-up according to the CTC Reporting and Data System (C-RADS E0–E4). The reference findings comprised the combined information from CTC (ULD, SD and contrast-enhanced CTC series) and a 4-year radiological and clinical follow-up. For the overall detection of reference findings (E2–E4) we found a statistically significant difference in favour of SD. This, however, was not the case when looking at classification of possibly important/important reference findings (E3–E4). Our results suggest that CTC with ULD (0.9 mSv) is comparable to SD (3.6 mSv) for identification of clinically relevant extracolonic pathology, but there is a large inter-observer variability.

## INTRODUCTION

Colorectal cancer (CRC) is the fourth most common form of cancer in Sweden after breast cancer, prostate cancer and squamous cell carcinoma/other forms of skin cancer. Approximately 6800 new cases are diagnosed yearly, 69% in the colon and 31% in the rectum^(1)^. Once clinical suspicion of CRC is aroused, diagnosis is usually verified by optical colonoscopy (OC) or by radiological techniques. For many decades, the radiological alternative to OC was barium enema examination, but in recent years computed tomography colonography (CTC) has, due to its higher diagnostic accuracy, gained increased acceptance as a complement to OC, and it has been recommended that barium examinations should no longer be performed in the work up of suspected CRC^(2)^.

Since most CRC develops from precursors in the form of polyps, screening has, by finding and removing these polyps, the potential to prevent cancer to develop. Screening can be performed by testing faecal samples for the presence of blood, by OC or with CTC.

In contrast to OC, CTC has the ability to reveal pathology outside the colon and rectum. Extracolonic CTC findings are commonly classified according to their clinical importance, as suggested by Zalis *et al*.^(3)^, who introduced the CTC Reporting and Data System (C-RADS) evaluation system, including classification of extracolonic findings as E1–E4, depending on the clinical relevance of the findings. Most such extracolonic findings are of no or limited clinical importance, but clinically significant lesions increase with patient age and occur in ~20% (E3) and 4.5% (E4) of patients investigated with CTC for clinical bowel symptoms^(4)^. While identification of colorectal lesions in the gas-filled bowel at CTC requires only limited radiation dose^(5)^, the detection of abnormalities in extracolonic soft tissues, where differences in density between normal and abnormal tissues usually are much smaller, requires more radiation. However, there is a general trend in abdominal computed tomography (CT) to lower the radiation doses, leading to the risk of a trade-off between diagnostic capability (image noise) and patient radiation dose. The purpose of this study was therefore to compare ultra-low-dose (ULD) CTC with standard dose (SD) CTC in terms of detection and characterisation of extracolonic lesions according to C-RADS (clinical relevance), degree of observer confidence and inter-observer variation.

Since evaluation of extracolonic findings is an integral part of CTC, it is of utmost importance to determine if CTC with reduced radiation dose leads to impaired capability to identify important extracolonic findings. It is also of interest to find out if it leads to false positive findings, which in turn may lead to unnecessary follow-up examinations, increased costs and patient anxiety, not least in a screening scenario.

## MATERIALS AND METHODS

### Ethical approval

The study protocol was approved by the Regional Ethical Review Board in Gothenburg (Dnr 649-05 and Dnr T 387-10), and written informed consent was obtained from all patients.

### Study population

During a 7-month period, 51 patients admitted for OC because of clinical suspicion of CRC (rectal bleeding or iron deficiency anaemia and/or positive faecal occult blood test) were included in a prospective study comparing the diagnostic outcome of CTC, as compared with same-day OC. CTC was performed with SD and ULD in each patient. The results concerning various aspects of colorectal lesion detection accuracy at CTC, including low-dose CTC, have been published earlier^(5)^. The present study concerned detection and characterisation of extracolonic lesions at SD and ULD in the same patient material. Due to lack of complete reference standard (no series with intravenous contrast medium), two patients were excluded from the present analysis, resulting in 49 study cases (18 men, 31 women) with a mean age of 67.1 years (range 51–86 years).

### CTC examination

CTC was performed on a 64-slice CT system (Lightspeed VCT, GE Healthcare, Chalfont St. Giles, UK) after routine bowel cleansing with an oral laxative (polyethylene glycol). Large bowel distension was obtained by rectal insufflation of carbon dioxide, administered by an automatic gas insufflator, according to the local routine. Intravenous administration of Buscopan (20 mg) or glucagon (1 mg) was used for bowel relaxation except in two patients with contraindications.

CT of the entire abdomen and pelvis was performed (at least covering the distance from the dome of the diaphragm to the symphysis pubis) initially in the supine position at standard X-ray tube current (SD) immediately repeated at ultra-low X-ray tube current (ULD) in the same body position. The patients were then examined in prone position using SD, 75 seconds after the start of an intravenous contrast medium injection (Visipaque 320 mg I/ml, injection rate 2.8 ml/sec).

### CTC scan parameters

The CTC scan parameters were collimation 64 × 0.625 mm; 0.625 mm slice thickness; reconstruction interval 0.625 mm; pitch 0.984; tube rotation time 0.5 second; tube voltage 120 kV and automatic *x*-, *y*- and *z*-dose modulation. A predefined tube current setting of 40–160 mAs for SD and 10–50 mAs for the ULD was used.

### Effective dose assessment

The potential risk due to radiation exposure was calculated using the formula^(6)^:

$$ E={E}_{\mathrm{DLP}}\times \mathrm{DLP}\left(\mathrm{mSv}\right), $$

where *E* is the effective dose, DLP is the dose-length product, *E*_DLP_ is a region-specific effective dose conversion coefficient. The CT system automatically calculated the DLP for each examination. Using this formula the mean effective dose was estimated to 3.6 ± 1.2 mSv for the SD examinations and 0.9 ± 0.4 mSv for the ULD.

### Observers

All examinations were independently evaluated by five consultant radiologists with expert knowledge in abdominal radiology, clinical experience 4–24 years.

### ViewDEX image evaluation

For the evaluation thin trans-axial (0.625 mm from all but one ULD series where only 1.3 mm were available) images from all examinations were retrieved from the Picture Archiving and Communication System, anonymized and re-reconstructed into 5/5 mm (thickness/increment) trans-axial and 4/3 mm coronal and sagittal images. The two re-reconstructed non-contrast series, ULD and SD prone series, were then separately fed into the ViewDEX evaluation system (Viewer for Digital Evaluation of X-ray images)^(7–9)^. ViewDEX is a DICOM-compatible software tool that can be used to display medical images with simultaneous registration of the observer’s response. ViewDEX allows the user to, on the screen, analyse X-ray images (scroll the images, measure Hounsfield values, measure distances, use different window settings, mark pathology and answer questions displayed on the same screen. Trans-axial, coronal and sagittal images were arranged in a single stack, after one another, and could be reached by scrolling through the image stack.

The ViewDEX evaluation systems were placed in secluded radiology reading rooms normally used for clinical routine reading, with similar ambient light approved for clinical routine reading. The images were evaluated on DICOM-calibrated medical grade displays. No time limits for reading were given; the observers had half or full working days set aside until they had finished all evaluations and were free to plan their own time. They all had oral instructions how to handle the ViewDEX system, and in addition, they all had the same written instructions for the image evaluation protocol. Initially the observers evaluated the unenhanced ULD series, blindly (without knowledge of patient identity or clinical information) and in a different random order for each observer. After a stipulated time span of no less than 6 weeks (median 35 weeks, range 9–134), the unenhanced SD series was then evaluated, again blindly and now in a new random order.

### Image evaluation protocol

Evaluation was based on a pre-designed examination protocol for assessment of extracolonic pathology per organ/tissue, including a confidence scale and an assessment of the need for follow-up according to the C-RADS^(3)^. Extracolonic findings are classified in the C-RADS according to clinical importance. A description of the C-RADS E-classification is given in Table 1.

**Table 1:** Definition of extracolonic CTC findings (E0–E4) according to C-RADS^(3)^.

E0	Limited examination, evaluation severely limited
E1	Normal examination or anatomic variations, no extracolonic abnormalities visible.
E2	Clinically unimportant finding and no work-up indicated.
E3	Probably unimportant finding, incompletely characterised. Work-up according to local traditions, clinical setting and patient preference.
E4	Potentially important finding, work-up recommended.

Suspected pathology was marked with the cursor on the screen and these markings were logged regarding Case ID, slice number and *x*- and *y*-coordinates together with the observer’s assessment of diagnostic certainty (1 = possible finding, 2 = probable finding, 3 = certain finding), kind of finding (C = cystic, S = solid, A = other, e.g. abdominal aortic aneurysm) and classification according to the C-RADS E-classification system (E0–E4).

The observer was not able to move to the next case until all parts of the examination protocol had been completed. If no pathology was found, this too had to be ticked off in order to make sure that no organ/part of the abdomen was overlooked. The resulting log files were later retrieved and all observer markings analysed as to location in indicated organ/structure and conformity to reference pathology.

### Reference standard

The reference standard was compiled by reading the original radiology report, reevaluating the full CT examinations, including the supine ULD and SD series, and the intravenous contrast medium enhanced prone series. In addition, a 4-year radiology and medical file follow-up was performed. Reference findings were discussed between members of the research group in order to reach a consensus regarding what pathology should constitute the reference standard and how to classify them according to the C-RADS E-classification system.

The primary goal was to evaluate soft tissue findings and therefore skeletal findings (such as degenerative changes, healed rib fractures, skeletal densities, etc.), minor or moderate arterial wall calcifications (as expected for age) and minor lamellar atelectasis in the lung bases were all excluded. Also findings in anatomic areas depicted on only one of the image series (ULD or SD) were excluded.

### Evaluation of data

Two types of evaluations were made. The first evaluation focused solely on the detection of lesions and disregarded the C-RADS E-classification. All markings corresponding to stated reference findings E2–E4, regardless of which E-class they were given by the observer, were classified as lesion localisations (LL) whereas markings not corresponding to reference findings were classified as non-lesion localisations (NL). The second evaluation focused on classification, taking into account both confidence level and E-classification. The objective was to determine how well the observers could distinguish lesions that require follow-up (E3 and E4 lesions according to the reference) from lesions that do not require follow-up (E1 and E2 lesions). All markings of E3–E4 reference findings also graded as E3 or E4 by the observers were classified as LLs, whereas if unmarked or marked as E1–E2 they were classified as NLs. E1–E2 reference findings marked as E3–E4, as well as markings not corresponding to reference findings, were classified as NLs.

**Table 2:** Description of the reference findings in the current study and their numbers for C-RADS categories E3 and E4^(3)^.

E3	No.	E4	No.
Unclear low-attenuating liver findings ≤5 mm	5	Low-density mass in the liver	2
Solid uterine mass	4	Large solid adrenal mass	2
Hiatal hernia	3	Gallbladder wall pathology	2
Suspected liver hemangioma	3	Pancreatic head mass	2
Enlarged adrenal	3	Solid mass right ovary	1
Ovarian cyst ≥2 cm	2	Solid mass dorsal to the ventricle	1
Pancreatic calcifications	2	Low-attenuating mass uncinate process	1
Dilated intrahepatic bile ducts	1	Mesenteric venous congestion	1
Splenomegaly	1	Solid mass near the pancreas	1
Dilated pancreatic duct	1	Solid mass upper abdomen	1
Dilated CBD	1	Large solid uterine mass	1
Pancreatic atrophy	1	Ventricular wall mass	1
Ascites	1	Solid mass/lymph nodes mesenteric root	1
Mesenteric fat stranding	1		
Aneurysm at the coeliac trunk	1		
Enlarged lymph nodes	1		

### Statistical methods

Based on the observer findings, free-response receiver operating characteristics (FROC) and alternative FROC (AFROC) curves were created using the jackknife alternative FROC (JAFROC) software^(10)^. In the FROC analysis, the lesion localisation fraction (LLF—proportion of detected pathology) is plotted as a function of non-lesion localisation fraction (NLF—average number of false markings per case) for different observer confidence levels^(11)^. For the *y*-axis (LLF) there is a maximum value of 1.0, whereas the *x*-axis (NLF) has no upper limit. In the AFROC analysis the LLF is plotted against the false positive fraction, obtained using the inferred ROC rating method where the rating of the highest rated NL on a case is used as the FP rating for the case^(12)^. The area under the AFROC curve was used as the primary figure-of-merit (FOM) of the JAFROC analysis^(13)^.

In the second evaluation (classification), the JAFROC variant including NLs from normal cases only was used, as recommended^(12)^. In the first analysis (detection), however, the JAFROC variant including NLs from all cases was used (JAFROC1), due to the very low number of normal cases for this analysis. Both evaluations treated observers and cases as random effects, enabling a generalisation of the results to the population of observers and cases.

## RESULTS

The reference standard contained 95 E2 findings (in 40/49 patients, 82%), 31 E3 findings (in 22/49 patients, 45%) and 17 E4 findings (in 12/49 patients, 24%). A detailed description of E3 and E4-reference pathology is given in Table 2.

Out of the 48 reference findings graded as E3 or E4, 14 were not marked by any of the observers. Out of the remaining E3 reference findings, two were only marked by one observer and six were marked by two observers. For the E4 reference findings one was marked by only one observer and five were marked by two observers.

### Evaluation time

The mean evaluation time, excluding all observation times exceeding 30 minutes indicating that the observer had been interrupted, resulted in an overall mean evaluation time of 11 minutes, range 7–17 minutes. For the ULD the corresponding evaluation times were 11 minutes, range 6–17 minutes and for the SD 11 minutes, range 6–17 minutes.

### Observer detection of reference findings

The observer detection of reference pathology was lower for ULD than for SD for E2, E3 and E4, details are given in Table 3. An average of 31 (range 20–37) out of the 95 E2 reference findings were marked as E2, E3 or E4 by one or more of the observers on the ULD examination and 43 (range 26–57) were marked on the SD. Twenty-five (range 17–31) reference findings were marked by the observers on both ULD and SD. Ten of the 95 (11%) E2 reference findings were not marked by any observer regardless of radiation dose.

**Table 3:** Detection (marking) of E2–E4 reference findings adding all observer markings from the five observers for ULD and SD (mean and range)

Observer 1–5	E2 (*n* = 95)	E3 (*n* = 31)	E4 (*n* = 17)
ULD	30.6 (20–37)	8.8 (3–12)	6.6 (4–10)
SD	42.6 (26–57)	11.4 (5–15)	8.2 (5–13)
Difference SD−ULD	12.0	2.6	1.6

**Table 4:** Agreement in E-classification of marked reference findings (LLs) on ULD and SD adding all markings from the five observers [mean number and (percentage)]

Observer 1–5	E1 (average)		E2 (average)		E3 (average)		E4 (average)	
Reference findings	ULD	SD	ULD	SD	ULD	SD	ULD	SD
E2-findings	0.4 (1.3%)		14.2 (46.4%)	19.0 (44.6%)	11.0 (35.9%)	19.2 (45.1%)	5.0 (16.3%)	4.4 (10.3%)
E3-findings			3.4 (38.6%)	2.6 (22.8%)	2.6 (29.5%)	4.8 (42.1%)	2.8 (31.8%)	4.0 (35.1%)
E4-findings			1.2 (18.2%)	1.6 (19.5%)	1.6 (24.2%)	1.8 (22.0%)	3.8 (57.6%)	4.8 (58.5%)

Nine (range 3–12) out of 31 E3 reference findings were marked as E2, E3 or E4 by one or more of the observers on the ULD examination and 11 (range 5–15) were marked on the SD examination. Seven (range 2–11) E3 reference findings were marked on both ULD and SD. Eleven of the 31 (35%) E3 reference findings were not marked by any observer regardless of radiation dose.

Seven (range 4–10) out of 17 E4 reference findings were marked as E2, E3 or E4 by one or more of the observers on the ULD examination and 8 (range 5–13) were marked on the SD examination. Six (range 4–7) reference findings were marked on both ULD and SD. Three of the 17 (18%) E4 reference findings were not marked by any observer regardless of radiation dose.

### Observer C-RADS E-classification of reference findings

Reference E2 findings correctly identified by the observers were overrated as E3 or E4 by the observers, both with ULD (52%) and SD (55%), as compared with the reference standard. Reference E3 findings were misclassified as E2 or E4 in 70% and 58% with ULD and SD, respectively, whereas reference E4 findings were underrated as E3 or E2 by the observers in 42% with ULD and 42% with SD. Details on classification are given in Table 4. There was no apparent difference in observer confidence level for reference findings (E2, E3 and E4) marked by the observers on both ULD and SD examinations. Numbers are given in Table 5.

**Table 5:** Description of the difference in confidence level between ULD and SD observer markings of the reference findings that were marked on both ULD and SD. Total number for all observers are given

Confidence level as compared with SD	Observer E2-findings	Observer E3-findings	Observer E4-findings	All observer findings
Unchanged at ULD	105	31	23	159
Increased at ULD	11	2	3	16
Reduced at ULD	14	3	2	19

### Markings of pathology not corresponding to reference findings (NLs)

The number of observer markings not corresponding to reference findings was 53; classifications for each observer for the two dose levels are presented in Table 6. Altogether those findings got 65 individual markings as possible pathology by the observers. Suspected pathology classified as E3 (follow-up according to local tradition) and E4 (follow-up recommended), not corresponding to reference findings were marked 18 times on the ULD series and 21 times on the SD series.

**Table 6:** Number of NLs (observer markings not corresponding to reference findings) and their classifications for each observer for the two dose levels ULD and SD, including all confidence levels

Observer	ULD				SD				
	E1	E2	E3	E4	E1	E2	E3	E4	
1	0	1	2	0	0	4	3	0	10
2	0	4	2	0	0	2	0	1	9
3	0	6	0	6	0	1	2	1	16
4	2	0	2	2	0	3	6	2	17
5	0	0	2	2	0	3	3	3	13
Total number	2	11	8	10	0	13	14	7	65

### Misclassification of reference findings (NLs)

E2 reference findings incorrectly classified as E3 or E4, regarded as NLs in the second JAFROC analysis, were common appearing 151 times on ULD and 48 times on SD as presented in Table 7.

**Table 7:** Number of E2 reference findings classified as E3 or E4 findings for each observer for the two dose levels ULD and SD, including all confidence levels

Observer	ULD		SD		
	E2 called as E3	E2 called as E4	E2 called as E3	E2 called as E4	
1	9	12	1	0	22
2	7	12	8	0	27
3	4	22	3	1	30
4	25	33	9	15	82
5	10	17	5	6	38
Total number	55	96	26	22	199

### JAFROC analysis: detection of reference findings

**Figure 1:**
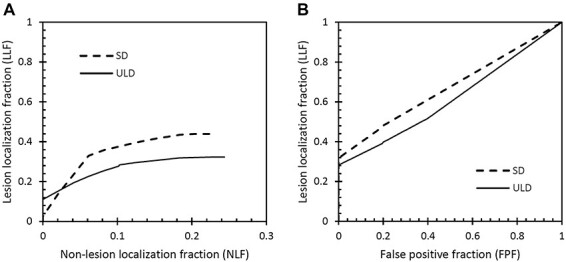
FROC curves (**A**) and AFROC curves (**B**) for the detection task (detection of E2-E4 reference findings) for the two dose levels SD and ULD.

FROC and AFROC curves for the detection of reference findings are presented in Figure 1. The JAFROC analysis showed a statistically significant difference in the area under the AFROC curve in favour of SD compared with ULD: the JAFROC FOM was 0.62 [95% confidence interval (CI): 0.57–0.67] for ULD and 0.67 (95% CI: 0.60–0.74) for SD (*p* = 0.02).

### JAFROC analysis: classification of reference findings

**Figure 2:**
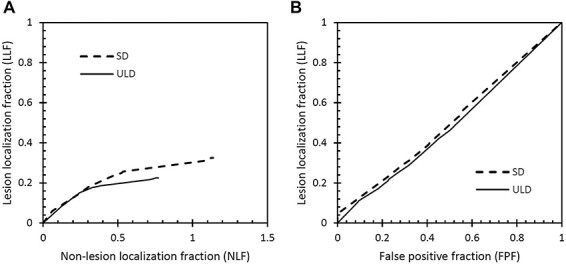
FROC curves (**A**) and AFROC curves (**B**) for the classification task (detection of E3–E4 reference findings) for the two dose levels SD and ULD.

FROC and AFROC curves for the classification task (detection of E3–E4 reference findings) are presented in Figure 2. The JAFROC analysis revealed no statistically signinficant difference in the area under the AFROC curve between ULD and SD: the JAFROC FOM was 0.48 (95% CI: 0.39–0.58) for ULD and 0.50 (95% CI: 0.41–0.60) for SD (*p* = 0.72).

## DISCUSSION

Evaluation of extracolonic findings is an integral part of CTC. Our aim was therefore to find out if ULD led to impaired capability to identify important extracolonic findings or if it led to false positive findings, which in turn potentially might lead to unnecessary follow-up examinations and patient anxiety.

In this study we looked at SD and ULD non-enhanced CTC images being obtained in the same body position and immediately following each other, allowing us to compare detection and characterisation of soft tissue pathology outside of the colon. Our study population comprised patients over 50 years of age with a clinical suspicion of colorectal disease, i.e. not a screening population. As per the reference standard, E2 findings were present in 82% of the patients, E3 findings in 45% and E4 findings in 24% of the patients. The number of E2 findings is in conformity with previous studies but somewhat higher regarding E3 findings and substantially higher regarding E4 findings than in some other studies^(3,14)^. However, there is a wide variation in reported frequencies, depending on e.g. patient selection (symptomatology, proportion of patients with malignancy), patient age and gender distribution, use of contrast enhancement, size of study group and definition of significant pathology^(15–19)^.

First, we analysed if there was a difference between ULD and SD in detection of reference pathology, regardless of C-RADS E-classification. We noticed that a large proportion of reference findings were not marked by the observers regardless of dose level. Thus, 11% of E2, 35% of E3 and 18% of E4 reference findings were not marked by any of the observers. To some extent, this was expected as the reference standard comprised not only the expert group assessment of the ULD and SD images, but also a global assessment including the contrast-enhanced image series and any follow-up studies for confirmation. Nevertheless, the results show that for the overall detection of reference findings, not taking into account what E-class they were given by the observers and looking at the whole group of observers, SD was statistically superior to ULD.

When analysing the clinically most relevant reference findings, i.e. findings classified as E3 or E4 that might or should be followed up, the difference between SD and ULD did not reach statistical significance. E3 reference findings were classified by the observers as E2 slightly more often with ULD, whereas E4 reference findings were classified as E2 in nearly 20% with ULD as well as SD. Reference E2 findings correctly identified by the observers were overrated as E3 or E4 by the observers, both with ULD (52%) and SD (55%), as compared with the reference standard. This, and a number of false positive observer findings (18 E3 and 21 E4), i.e. without corresponding reference findings, would potentially, according to work-up recommendations, result in a substantial number of unnecessary follow-up examinations and possibly related patient anxiety.

However, there was a large variation in detection rate between the five observers, with ULD as well as SD. To our knowledge, the inter-observer variation in E-classification has not previously been analysed. The inter-observer variation may have several reasons, such as different levels of clinical experience, different experience with the C-RADS E-classification system and varying opinions amongst the observers about the clinical relevance of extracolonic findings.

Comparing the difference in level of confidence for observer markings that were given a level of confidence both on ULD and SD showed no substantial difference, most findings were given the same level of confidence. Thus, the increased image noise with ULD does not seem to create a higher level of uncertainty, as compared with SD.

One might have suspected that the observation times for the ULD should be longer than for the SD since the images are presented with a higher noise, and some of them according to the C-RADS E-classification even might have been called as E0—non-diagnostic, but we found no difference in time used for the evaluations, mean time used for both ULD and SD was 11 minutes, range 6–17 minutes. This indicates that the observers did not feel greater hesitation in evaluation of ULD than of SD images.

We used the terms ULD and SD patient radiation dose, and the estimated mean effective dose levels were 0.9 and 3.6 mSv for ULD and SD, respectively. The terms SD and ULD have no universal definition, but our radiation dose levels are in approximate agreement with those of others in CTC^(20)^ and urinary low-dose CT examinations^(21)^. In a study by Lambert *et al*.^(22)^ it was shown that CTC with hybrid iterative reconstruction (HIR) and iterative model reconstruction techniques were suitable for sub-mSv ULD CTC without sacrificing diagnostic performance regarding colonic findings. For the ULD–HIR there were significantly fewer extracolonic findings compared with SD but for the C-RADS E4 category the detection rate was similar. In another study^(23)^ it was stated that 1-mSv CTC was not feasible using standard filtered back projection (FBP), but diagnostic performance could be improved by using iterative reconstruction (IR) algorithms. Vardhanabhuti *et al*.^(24)^ found no difference in detection of extracolonic findings comparing adaptive statistics and model-based iterative reconstruction CTC, but they used higher effective dose levels. The examinations used in the present study were performed using FBP, which could have an impact on our results evaluating extracolonic findings. In previous studies^(5)^, however, FBP examinations have proven sufficient for evaluating colonic pathology, which is the primary focus of CTC. As extracolonic pathology is not the primary focus, it seems reasonable to accept a slightly suboptimal extracolonic detection rate, especially when radiation dose is an issue, as in younger patients or in a CRC screening scenario.

There are limitations to this study. First, the number of cases, and thereby the number of clinically relevant extracolonic findings, was limited. However, each patient case served as its own control, with ULD and SD performed in the same body position in the same session, and the limited case number was partly compensated for by the use of multiple (five) observers. Second, the reference standard was based on consensus expert opinion, which may be affected by bias, and surgical or histopathological information was not available in all cases, although 4-year radiology and medical file follow-up was done to confirm and classify the reference findings. Third, the study was performed separately from the clinical routine and does therefore not necessarily mirror the clinical situation. Fourth, our study was based on symptomatic patients, and therefore, it is difficult to generalise our findings to a screening population, where the frequency, type and implication of extracolonic findings may be different. Fifth, recall bias is a well-known confounder in comparative imaging studies, especially with a material limited in patient numbers and with limited pathology. We prescribed a 6-week interval between reading sessions of ULD and SD, and the actual interval was median 35 weeks. We also tried to minimise recall bias by blinding of patient data and by random reading order.

In clinical routine, ULD CTC has proven to be acceptable for the diagnosis of colonic wall pathology^(5)^. The large number of missed extracolonic pathology as well as a number of false positives in our study, both on ULD and SD, however, indicates that for an adequate assessment of extracolonic soft tissue pathology SD examination with intravenous contrast is necessary. This ends up in a trade-off between diagnostic accuracy, radiation dose and cost, which may be different in different situations, such as a clinical or screening scenario.

## CONCLUSION

In this study, comparing ULD CTC with SD CTC, we found a statistically significant difference in favour of SD regarding overall detection of extracolonic soft tissue pathology. There was, however, no difference between ULD and SD when looking at the clinically most relevant findings, i.e. E3 and E4 lesions that would require follow-up. Using FBP CTC technique with ULD and a mean patient radiation dose of 0.9 mSv therefore seems comparable to SD with a dose of 3.6 mSv in this respect. Implementing the C-RADS E-classification system for extracolonic findings seems to be associated with considerable observer variation, which requires further analysis.
